# Rapid, Single-Step Monitoring of Monoclonal Antibody Bioavailability by Using a TNF-α-Based Multiepitope DNA Nanoswitch

**DOI:** 10.1021/acs.analchem.5c01239

**Published:** 2025-04-08

**Authors:** Denise Di Lena, Edoardo Sisti, Erik Brass, Erica Belforte, Bruna Marini, Alessandro Porchetta, Laura Squarcia, Eleonora Da Pozzo, Alessandro Bertucci, Rudy Ippodrino

**Affiliations:** † Ulisse BioMed Laboratories, Area Science Park, 34149 Trieste, Italy; ‡ Department of Chemistry, Life Sciences and Environmental Sustainability, University of Parma, Parco Area delle Scienze 17/a, 43124 Parma, Italy; § Department of Pharmacy, University of Pisa, via Bonanno 6, 56126 Pisa, Italy; ∥ Department of Chemistry, University of Rome, Tor Vergata, Via della Ricerca Scientifica, 00133 Rome, Italy; ⊥ CISUP, Center for Instrumentation Sharing of the University of Pisa, Lungarno Pacinotti 43/44, 56126 Pisa, Italy

## Abstract

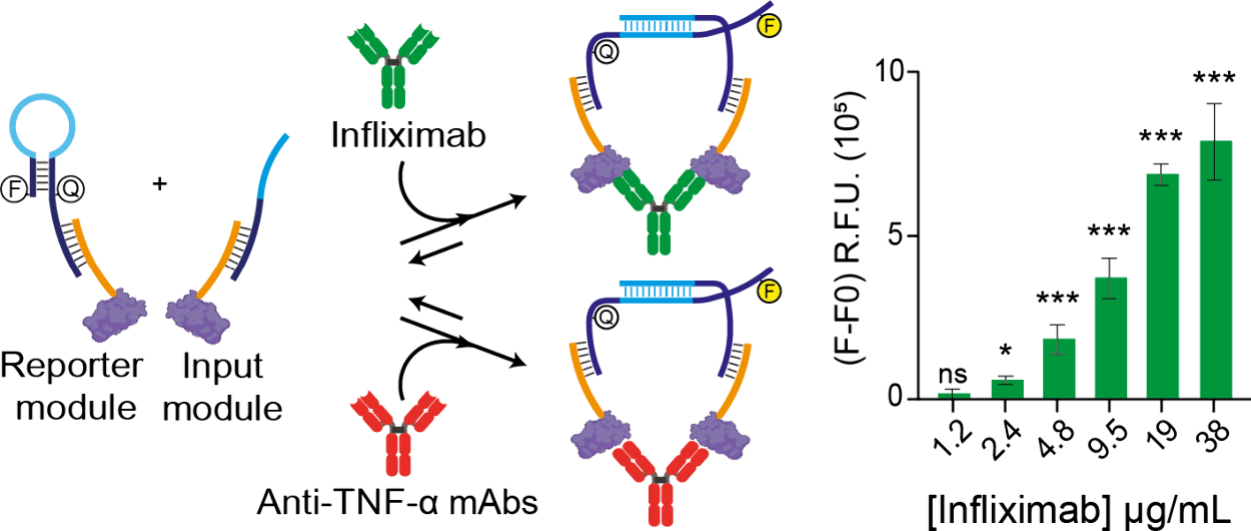

Therapeutic drug monitoring (TDM) is increasingly valuable for tailoring personalized therapy, particularly in managing chronic inflammatory diseases where overtreatment can cause significant side effects. Monoclonal antibodies (mAbs), a primary therapeutic approach for these conditions, face challenges from antidrug antibodies (ADAs), which can reduce mAb bioavailability and efficacy. To address these issues, we utilized Tumor Necrosis Factor α (TNF-α) as a binding moiety in a fluorescence-based programmable nanosensor within the NanoHYBRID (NH) platform developed by Ulisse Biomed S.p.A. By directly conjugating TNF-α to DNA probes, we developed a rapid, homogeneous, affinity-based assay capable of detecting multiple mAbs targeting distinct epitopes on the same protein. This NH platform effectively detected therapeutic concentrations of clinically relevant mAbs, such as Infliximab, Adalimumab, and Golimumab, in blood serum samples in a one-step process, bypassing the need for time-intensive washing steps. Moreover, the NH sensor exhibited heightened sensitivity to the presence of ADA, which impacted drug quantification, indicating its utility for monitoring bioavailable mAb levels. Compared to ELISA, the NH platform demonstrated superior sensitivity to ADAs, suggesting its potential as a highly specific, modular solution for TDM. This modular design allows the NH platform to create multiepitope nanosensors capable of measuring bioavailable mAbs in a single step.

## Introduction

Chronic inflammatory diseases represent a different group of conditions that persist for one year or more, often requiring continuous medical attention and significantly impacting daily life. The World Health Organization (WHO) recognizes these diseases as among the leading causes of global mortality.^1^ Conditions such as rheumatoid arthritis, Crohn’s disease, psoriasis, psoriatic arthritis, and ankylosing spondylitis share common pathogenic mechanisms, with tumor necrosis factor-alpha (TNF-α) playing a central role.^2,3^ TNF-α is a pleiotropic cytokine produced as a 26 kDa transmembrane protein^4^ that is predominantly active in its 17 kDa soluble form. This soluble TNF-α interacts with its receptors TNFR-1 and TNFR-2 to regulate bioactivity. Although TNF-α provides protective effects against infections, its overexpression can contribute to chronic inflammation, leading to progressive tissue damage in these diseases.^5−7^ Biological therapies, particularly monoclonal antibodies (mAbs) targeting TNF-α, have been developed to interrupt TNF-α–receptor interactions and mitigate inflammatory responses.^8^ Infliximab, the first anti-TNF-α mAb, paved the way for the development of other biologic agents, such as Adalimumab, Certolizumab, and Golimumab, with each agent exhibiting specific actions due to its distinct pharmacological properties.^9,10^ The binding sites of these antibodies have been studied intensively, revealing that, while all these antibodies target TNF-α, they bind and recognize different epitopes of the TNF-α protein.^11−15^ Despite their clinical efficacy, not all patients respond to biological therapies. Some patients experience a primary lack of clinical response, while up to 50% experience a secondary loss of efficacy over time, leading to therapeutic discontinuation or side effects.^16^ Nonresponse can result from suboptimal drug levels, idiopathic factors, or the development of antidrug antibodies (ADAs).^17^ ADAs are generated when the immune system recognizes specific portions of the biological drug as foreign, often within the first 2 weeks of treatment.^18^ These ADAs can interfere with drug function through two primary mechanisms: pharmacokinetic interference, where ADAs trigger an increase of drug clearance, and pharmacodynamic interference, where ADAs, by binding to the drug, prevent drug–target interactions, leading to therapeutic failure.^18−21^ Therapeutic drug monitoring (TDM) has become an essential tool in precision medicine, allowing for individualized treatment adjustments to optimize drug efficacy while minimizing side effects and costs.^22,23^ Although current TDM methodologies, such as enzyme-linked immunosorbent assays (ELISA), are widely used to measure biologic drug or ADA levels, they have significant limitations. These techniques are often complex, requiring multiple washing and incubation steps, and necessitate specialized personnel.^24^ Consequently, there is a growing demand for more efficient methods for monitoring the biologic drug concentration levels. To address the limitations of conventional technologies, recent advancements in biomolecular platforms have focused on the rapid, one-step detection of mAbs. These technologies often leverage DNA nanotechnology to create biomolecular probes that undergo conformational changes upon mAb binding, generating a measurable output.^25−30^ One such platform, developed by Ulisse BioMed S.p.A., is the NanoHYBRID (NH) platform. This innovative technology employs structure-switching DNA probes that detect mAbs through a homogeneous sensing mechanism, providing a rapid and accurate measurement of drug levels, with the potential to enhance recovery rates and reduce treatment costs, particularly for severe conditions such as cancer and chronic diseases.^31^ Specifically, the NH platform uses hybrid DNA/PNA-peptide nanoswitches that produce a fluorescent signal within minutes when antibodies bind, facilitating rapid and accurate detection of specific antibodies directly in blood serum.^31^ To date, the NH platform has been designed by featuring small peptides (single-epitope) as recognition elements. However, the platform’s modularity allows for the replacement of the PNA-peptide probe, which can only bind to one specific mAb, with a ssDNA–whole protein conjugate, enabling the detection of multiple mAbs targeting different epitopes on the same protein. This multiepitope strategy provides the foundation for developing a novel detection technology for mAbs targeting different epitopes displayed on the same protein and allows for the simultaneous investigation of ADA interference with drug measurements. By eliminating the time-consuming steps required in traditional methods, this approach has the potential to advance TDM practices, improving outcomes for patients with chronic inflammatory diseases.

## Experimental Section

### Oligonucleotides, Monoclonal Antibodies, and Proteins

ssDNA modified with AlexaFluor 680 and Black Hole Quencher 2 (no. 1, MB) was purchased from MultiplexDX Int. (Bratislava, Slovakia). Peptide nucleic acid (PNA)–peptide chimeric probe was purchased from HLB Panagene Co.LTD (South Korea). Input Strand (#2) was purchased from Metabion Int. AG (Planegg, Germany). ssDNA oligonucleotide used for the conjugation step, modified with DBCO (dibenzocyclooctyne) at the 3′ end, was purchased from Metabion Int. AG (Planegg, Germany). TNF-α (17.4 kDa) was purchased in lyophilized form from ACROBiosystems Inc. (USA) and reconstituted in HyClone HyPure (Cytiva Europe GmbH, Freiburg, Germany), molecular-grade water at 2 mg/mL. Infliximab, Adalimumab, Etanercept, Certolizumab, Golimumab, and Trastuzumab were purchased already reconstituted from Evidentic GmbH (Berlin, Germany). Anti-Infliximab Antibody HCA233 (binding ADA) and Anti-Trastuzumab Antibody HCA176 (binding ADA) were purchased already resuspended from Biorad Laboratories Srl. Anti-Infliximab Antibody HCA213 (neutralizing ADA) and Anti-Trastuzumab Antibody HCA177 (neutralizing ADA) were purchased and resuspended from Biorad Laboratories Srl.

## Nucleotide Sequences

Alexa680/BHQ2-modified DNA stem–loop (#1, MB): 5′-GTC ACC GCA AAA TAA GAT C(BHQ2-dT) C GCA CCT GAG TGG TAA TCT AGT GCG T (Alexa680)-3′.

Input strand (#2): 5′-TAG TCG TAA GCT GAT ATT TTT TTT TTT TTT TTT TTT TTA GAT TA CCA CTC AG-3′.

PNA–peptide (#3): 5′-TCT TAT TTT CGG GTG ACT TTT TTT TTT-3′-N-term–QLG PYE LWE LSH–C-term.

TNF-α oligo sequence (#4): 5′-ATC TTA TTT TGC GGT GAC TTT TTT TTT T-3′-TNF-α.

Infliximab-oligo sequence: 5′-ATC TTA TTT TGC GGT GAC TTT TTT TTT TTT TTT TAA AAT TTT TTT TTT T-3′- Infliximab.

### Protein–Oligonucleotide Conjugation

Oligonucleotide–TNF-α conjugates were obtained using an amine-coupling kit provided by Dynamic Biosensors GmbH, following the manufacturer’s guidelines, as reported here: (a) oligonucleotide activation; (b) protein addition and incubation at room temperature (RT) for 1 h and overnight at 4 °C; (c) purification using the *proFIRE* machine (Dynamic Biosensors GmbH) followed by buffer exchange in PBS (Phosphate Buffer Saline, Invitrogen). The conjugate was quantified using the NanoDrop 1000 spectrophotometer (Thermo Fisher Scientific, Monza, Italy) by measuring absorbance at λ = 260 nm and applying the following equation:
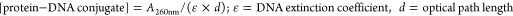


For each conjugation procedure, the reaction yield % was calculated using the following equation:
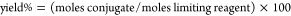


Reaction yield was calculated considering DBCO-modified ssDNA oligos as the limiting reagent, with a ratio of 1:2 (protein:ssDNA).

Conjugates could be immediately used or stored for a short period at a temperature between 0 and 4 °C or stored for longer periods at −80 °C after adding trehalose at 10% (V/V) as a cryoprotective agent. The same protocol was carried out to obtain a conjugate between 49 bp oligonucleotide and Infliximab, instead of the oligonucleotide-TNF-α.

### NanoHYBRID Platform

For each experiment, the required components of the NH platform were diluted in the NH reaction buffer [150 mM NaCl and 20 mM HEPES (4-(2-hydroxyethyl)-1-piperazineethanesulfonic acid purchased from Merck KGaA (Milan, Italy))] and reacted in a 25 μL well of black 384-well low-binding microplates (Greiner bio-one International GmbH, Germany), and all the steps were performed on ice. Initially, 5 μL of Alexa680/BHQ2-modified DNA stem-loop (50 nM) was mixed with 5 μL of TNF-α oligo sequence (500 nM), obtained from the previous conjugation, or 5 μL of PNA–peptide (250 nM) and 10 μL of antibody (250 nM). After sample loading, an initial spin of the plate was performed to ensure the proper mixing of all reagents. The plate was then incubated on ice for 10 min, protected from light. As a final step, 5 μL of an Input strand (150 nM) was added, followed by another spin of the plate. The fluorescent signal was measured using the EnVision high-throughput screening microplate reader (PerkinElmer Inc., USA) with an excitation filter at λ = 660 nm and an emission filter at λ = 720 nm, both with 90% transmittance and a cutoff mirror at λ = 585 nm. Measurements were performed at 25 °C. Data was collected after 30 min of incubation to ensure stabilization of the components away from light sources. Experiments in crude samples were performed using 10% final v/v commercially available standard human serum (Sigma-Aldrich). Signal gain is calculated based on the following equation:
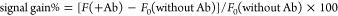
where *F*(+Ab) is the fluorescent signal obtained in the presence of the target antibody and *F*_0_(without Ab) is the fluorescence signal obtained in the absence of the antibody (background).

### ELISA Assay

Trastuzumab quantification was performed using a LISA TRACKER Duo Trastuzumab ELISA kit, a commercially available product, purchased from Theradiag, according to the manufacturer’s instructions.

### Statistics

Results are reported as the mean ± SD (standard deviation). Statistical significance was assessed using Student’s *t* test and one-way ANOVA test; *p*-values were used to determine significance levels.

## Results and Discussion

**Figure 1:**
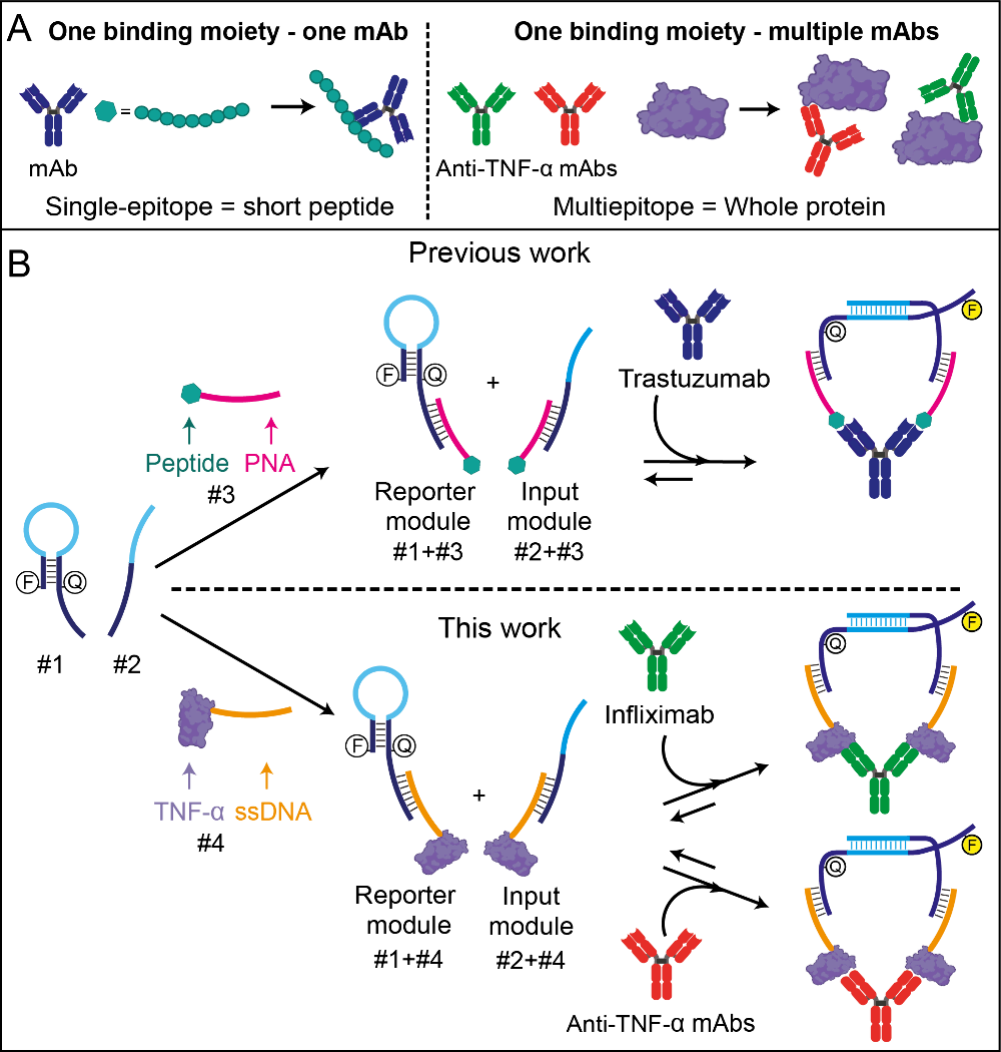
(A) Comparison between the previously established single-epitope NanoHYBRID platform, which enables detection of one target mAb at a time, and the newly developed multiepitope platform, which allows detection of multiple target mAbs simultaneously. The single-epitope platform utilizes a short peptide as the binding moiety recognizing Trastuzumab mAb, while the multiepitope platform employs a whole protein, displaying multiple epitopes for the detection of several target mAbs. (B) Schematic representation of the detection principle. Binding of the target antibody to the reporter (#1 + #3 or #1 + #4) and input modules (#2 + #3 or #2 + #4) induces colocalization, facilitating hybridization between #1 and #2, which generates a fluorescent signal proportional to the target analyte concentration.

Building on the previously established single-epitope DNA/PNA-based NanoHYBRID (NH) platform that was able to detect Trastuzumab drug,^31^ this study developed a multiepitope nanosensor capable of measuring a broad range of mAbs directed against TNF-α. The novelty of the presented multiepitope NH format lies in using the whole TNF-α protein as a nanosensor binding moiety, replacing the single-epitope PNA-peptide used in earlier designs (Figure1A). This approach enables a single binding moiety to expose all epitopes, allowing detection of various anti-TNF-α mAbs without the need to modify the binding component for each target, as required in previous designs. To achieve this, a TNF-α–ssDNA conjugate was synthesized using a commercial click chemistry protocol (Amine Coupling Kit 3, provided by Dynamic Biosensors GmbH, München, Germany; Figure S1). This protocol employs a dibenzocyclooctyne (DBCO)-modified ssDNA oligonucleotide and targets lysine residues on the protein possessing free amino groups (−NH_2_) available to form a covalent bond, ideally at a stoichiometric ratio of 1:1 DNA:protein. After conjugation, the TNF-α-DNA product was purified using the *proFIRE* system (Dynamic Biosensors GmbH). This conjugation step resulted in linking the binding moiety with the ssDNA oligonucleotide #4, which is partially complementary to the other NH components, oligonucleotides 1 and 2 (Figure 1B). Similar to the single-epitope nanosensor, the multiepitope nanoswitch operates through a colocalization effect triggered by the presence of target antibody (Figure 1B). The multiepitope nanoswitch is composed of three main components: (i) Molecular Beacon (no. 1, Figure 1B): This component is a fluorophore/quencher-modified DNA stem-loop sequence with a 17-bp single-stranded tail complementary to the TNF-α–DNA conjugate (no. 4, Figure 1B). The stem-loop structure results from a 5-bp self-complementary domain, bringing the fluorophore and quencher into close proximity and resulting in low fluorescence in the absence of the target. (ii) Input Strand (no. 2, Figure 1B): This ssDNA sequence is designed to invade the MB at its loop region (light blue section) through a 15-bp complementary region. It also contains a 17-bp sequence complementary to the TNF-α–DNA conjugate. (iii) Multiepitope DNA oligonucleotide conjugate (#4, Figure 1B): This core element is generated through a conjugation and purification process. It consists of a 28-base ssDNA sequence partially complementary to both the MB (#1, Figure 1B) and the Input Strand (#2, Figure 1B). The conjugated TNF-α protein serves as a binding site for multiple mAbs that target different epitopes on its surface. The TNF-α–DNA conjugate is designed to efficiently hybridize with the MB and the Input Strand, forming the Reporter Module (#1 + #4, Figure 1B) and the Input Module (#2 + #4, Figure 1B). In the absence of target analytes, hybridization is inefficient, resulting in a weak background fluorescence signal. However, when target mAbs are present, their interaction with the TNF-α–DNA conjugate brings the Reporter Module and the Input Module into close proximity. This leads to a significant increase in local concentration, promoting efficient hybridization and generating a highly fluorescent signal that is directly proportional to the concentration of the target mAb (Figure 1B).

**Figure 2:**
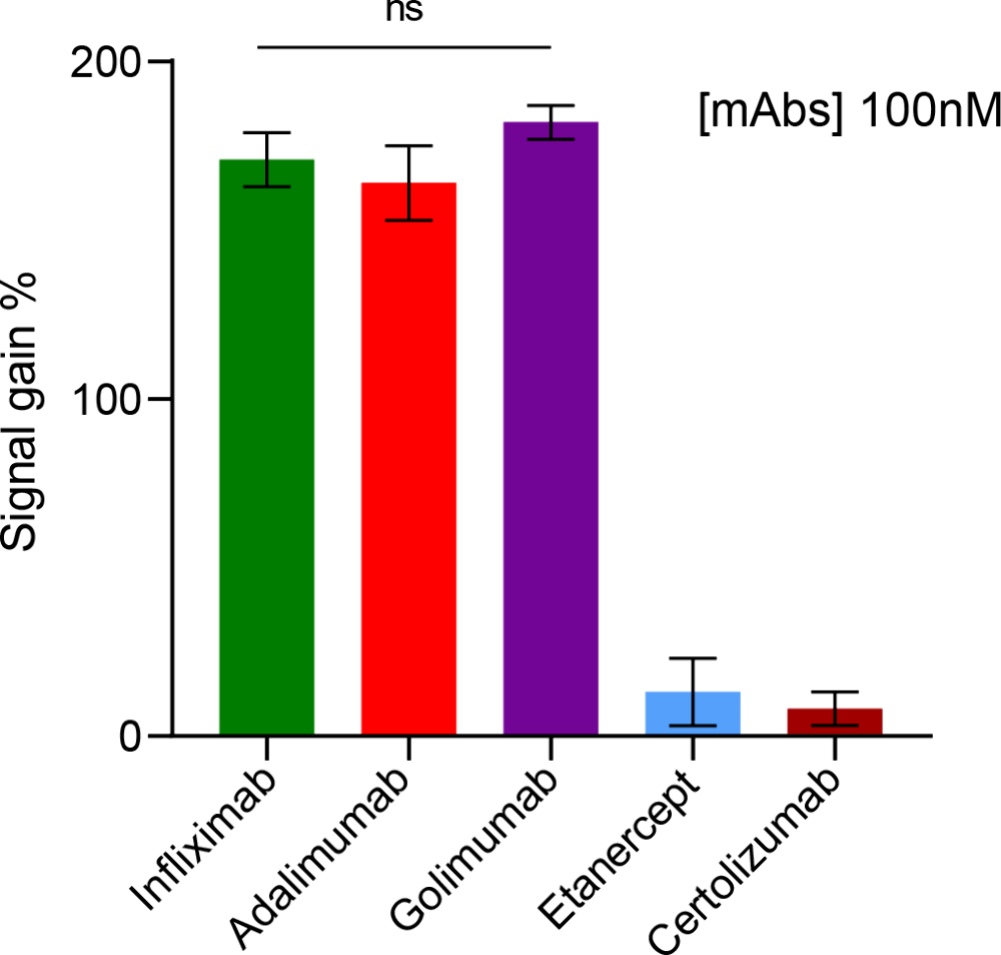
Multiepitope nanoswitch-based detection of various anti-TNF-α monoclonal antibodies (mAbs), specifically Infliximab, Adalimumab, and Golimumab, each at 100 nM (*n* = 3, mean ± SD).

Five TNF-α inhibitors, including Etanercept, Infliximab, Adalimumab, Certolizumab-pegol, and Golimumab, have been approved by the FDA for the treatment of inflammatory diseases.^11^ The multiepitope nanoswitch performance in detecting clinically relevant anti-TNF-α mAbs was assessed focusing on those five commercially available mAbs. These included three drugs that have two recognition sites for TNF-α at each of their arms, i.e., Adalimumab, Infliximab, and Golimumab (Figure 2). Etanercept and Certolizumab, the other two drugs targeting anti-TNF-α but having only one recognition site for TNF-α, were used as a control. It was hypothesized that the multiepitope sensor would not trigger the same response that is stimulated by binding to the two recognition sites. The nanosensor effectively identified Adalimumab, Infliximab, and Golimumab at this concentration, demonstrating its capability to detect these mAbs, producing a comparable signal with no significant differences observed. However, as expected, Etanercept and Certolizumab could not be detected. This discrepancy in detection has to be attributed to the unique structural conformations of these two TNF-α antagonists: Etanercept is a genetically engineered fusion protein consisting of two identical TNFR2 extracellular domains linked to the Fc fragment of human IgG1, while Certolizumab-pegol is a PEGylated (polyethylene glycol) Fragment antigen binding (Fab) of a humanized mAb that binds and neutralizes human TNF-α.^3,11,15^ These structural conformations influence their binding interactions with the TNF-α–DNA conjugate used in the nanoswitch.

**Figure 3:**
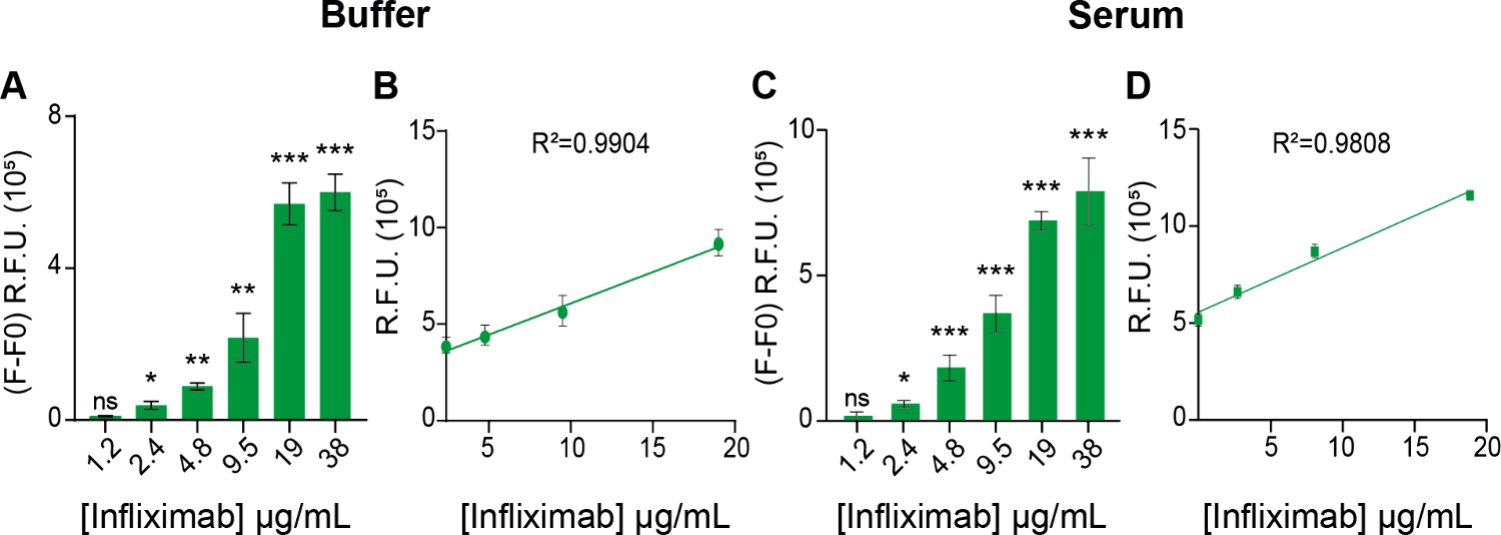
Multiepitope nanosensor analytical performance using Infliximab as the reference anti-TNF-α mAb. (A) Dose–response assay in spiked samples in NH buffer. The nanosensor demonstrated the ability to detect Infliximab at concentrations as low as 2.4 μg/mL. (B) Linearity range in NH buffer samples, ranging from 2.4 to 19 μg/mL. The curve is described by the following equation: *y* = (32500 ± 2259)*x* + (291022 ± 24751), *R*^2^ = 0.9904. (C) Binding dose–response assay in crude samples (undiluted blood serum). The nanosensor demonstrated the ability to detect Infliximab at concentrations as low as 2.4 μg/mL. (D) Linearity range in crude samples ranging from 2.4 to 19 μg/mL of Infliximab. The curve is described by the following equation: *y* = (37586 ± 1514)*x* + (465694 ± 16588), *R*^2^ = 0.9808. All graph bars report *n* = 3, mean ± SD. One-way ANOVA was performed. *p ≤ 0,05; **p ≤ 0,01; ***p ≤ 0,001; ns = not significant.

Dose–response experiments were then conducted by spiking increasing concentrations of Infliximab into both purified samples (NH Buffer) and crude samples (undiluted blood serum), with concentrations ranging from 0 to 38 μg/mL (Figure 3). Additionally, the performance of the multiepitope sensor was compared with that of a single-epitope nanosensor using Trastuzumab as the reference target mAb (Figure S2). The multiepitope nanosensor effectively detected Infliximab concentrations as low as 2.4 μg/mL and showed a clear response to varying concentrations in purified samples (NH buffer; Figure 3A). In these samples, the multiepitope sensor exhibited a linear detection range from 2.4 to 19 μg/mL, with *R*^2^ = 0.9904 (Figure 3B). This performance remained consistent when moving to crude samples (undiluted blood serum; Figure 3C,D), demonstrating the robustness of the nanosensor across different sample matrices. Importantly, the multiepitope sensor achieved a sensitivity comparable to that of the single-epitope sensor (Figure S2), accurately distinguishing between varying antibody concentrations without any performance loss, even in complex matrices, such as undiluted blood serum.

**Figure 4:**
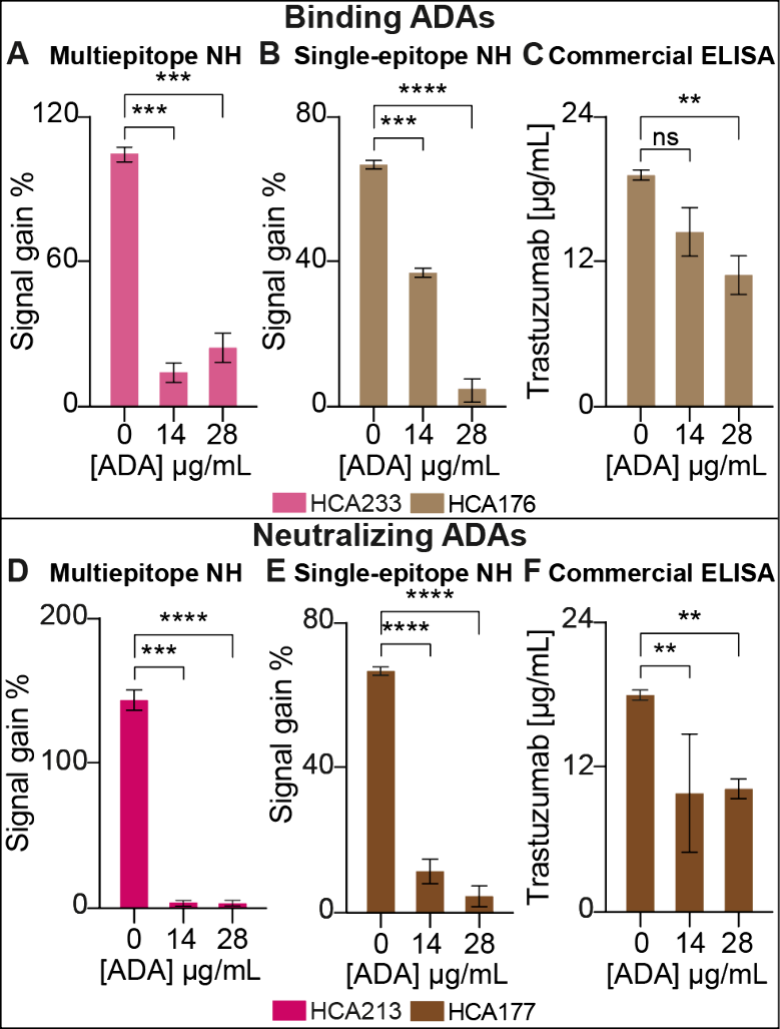
Effect of antidrug antibodies (ADAs) on drug quantification in the presence of a fixed amount of 15 μg/mL Infliximab and Trastuzumab. Multiepitope nanoswitch assays (A, D): The Infliximab-specific assay showed around 80% signal loss in the presence of binding ADA HCA233 and total signal loss with neutralizing ADA HCA213 at 14 and 28 μg/mL. Single-epitope nanoswitch assays (B, E): The Trastuzumab-specific assay showed around 50% signal loss with binding ADA HCA176 at 14 μg/mL, total loss at 28 μg/mL, and a similar pattern with neutralizing ADA. Commercial ELISA assays (C, F): The Trastuzumab ELISA showed no significant effect at 14 μg/mL of binding ADA and a 50% signal reduction at 28 μg/mL. In the presence of neutralizing ADA, it showed a 40% signal reduction at both 14 and 28 μg/mL. All graph bars report *n* = 3, mean ± SD. One-way ANOVA was performed. **p ≤ 0.01; ***p ≤ 0.001; ****p ≤ 0.0001; ns = not significant.

To investigate the effect of antidrug antibodies (ADAs) on drug quantification, a series of experiments were conducted using binding ADAs, HCA233 (an anti-Infliximab antibody) and HCA176 (an anti-Trastuzumab antibody), which interfere with pharmacokinetics by increasing mAb clearance and neutralizing ADAs, HCA213 (an ani-Infliximab antibody) and HCA177 (an anti-Trastuzumab antibody), which interfere with pharmacodynamics by preventing the mAb from interacting with its target molecules, thereby reducing its therapeutic efficacy. These experiments utilized the novel multiepitope nanosensor for Infliximab, the well-characterized single-epitope nanoswitch for Trastuzumab,^31^ and a commercial ELISA for Trastuzumab detection (Figure 4).

In purified samples with a fixed drug concentration of 15 μg/mL, two ADA concentrations of 14 and 28 μg/mL were introduced. A significant decrease in the detected concentrations of both Trastuzumab and Infliximab were observed in the presence of both types of ADAs, using the respective single- and multiepitope nanoswitches. The larger variation observed at 28 μg/mL binding ADA may be due to nonspecific interference effects at higher ADA concentrations. Regarding the differences between the multi- and single-epitope nanoswitches, we note that ADAs at 14 μg/mL have a more pronounced effect on the multiepitope system. This likely results from steric hindrance: ADAs binding to Infliximab impedes its interaction with TNF-α–DNA conjugates (a whole protein) to a greater extent than in the single-epitope system. These results suggest that the NH platform is highly sensitive to the presence of ADAs, demonstrating its capability of detecting only the bioavailable portion of the drug. In contrast, when analyzing the impact of ADAs on drug quantification using a commercial ELISA kit, minimal or no significant effect was observed. This highlights a key distinction between the NH platform and traditional ELISA assays, with the NH platform being more sensitive to the influence of ADAs on the drug bioavailability. Furthermore, by modifying the binding moieties of the multiepitope platform–specifically by conjugating Infliximab to a specific oligonucleotide–we demonstrated the ability to target and quantify HCA233, showcasing the platform’s potential to provide comprehensive information for TDM (Figure S3). This versatility is enabled by the general design of the NH platform, which allows for targeting diverse analytes by modifying the relevant binding moieties.^32^ In patients with chronic inflammatory diseases, maintaining adequate drug levels is critical for achieving optimal therapeutic outcomes, as insufficient levels can lead to suboptimal responses.^22,23^ This study developed a multiepitope sensor to address this need, enabling the direct detection of multiple mAbs targeting different epitopes on TNF-α in serum samples, thereby overcoming limitations of the previously available single-epitope DNA nanoswitch sensor. The NH platform also showed resilience in detecting mAb variations in crude samples, suggesting robustness comparable to that of single-epitope nanosensors. Although future improvements could expand its sensitivity and optimize precision, current findings support the NH platform as a practical solution for multiepitope mAb detection, which is especially beneficial for targeting proteins with complex or variable binding epitopes. While some therapeutics, such as Etanercept and Certolizumab, lack the structural attributes necessary for colocalization and detection by this nanosensor,^33^ the platform’s modularity allows potential adaptation for new biodrug classes, including nanobodies and bispecific antibodies. TNF-α antagonists, whose use is growing, may exhibit structural differences that should be evaluated case-by-case.^11,34^ Additionally, the NH platform’s performance in assessing ADA influence on drug quantification revealed that both single- and multiepitope nanosensors were highly sensitive to ADAs, showing a clear reduction in detectable drug levels in their presence. This indicates that the NH platform detects only the bioavailable portion of mAbs, distinguishing it from commercial ELISA, which includes ADA-bound drug complexes in its measurements, potentially overestimating active drug levels. The NH platform’s steric sensitivity to ADA interference highlights its advantage for bioavailability-focused quantification, offering a more accurate assessment of treatment efficacy and supporting precise TDM in therapeutic regimens. Understanding bioavailability is essential for determining the correct dosage, administration route, and therapeutic schedule.^35,36^

## Conclusion

TDM has become a critical element of precision medicine, particularly in addressing interindividual variability in the pharmacokinetics of numerous medications. In this context, the NanoHYBRID platform (Ulisse BioMed S.p.A) was designed for rapid and direct drug monitoring. This study leveraged the platform’s versatile and modular design to create a multiepitope nanosensor that allows for the simultaneous monitoring of several mAbs targeting the same binding moiety. The newly developed biosensor demonstrated its ability to detect three different mAbs commonly used in clinical settings for the treatment of immune-mediated chronic disease. Additionally, it effectively differentiated between various drug concentrations, paving the way for its application in patient sample testing for anti-TNF-α mAbs. Importantly, the study assessed the influence of ADAs on the NH platform, revealing a significant effect on drug detection. Comparative analysis with a commercial ELISA showed that the NH platform is more sensitive to ADA interference, thereby measuring only the bioavailable portion of the drug. This is a noteworthy finding, as different forms of ADAs can promote drug degradation without necessarily affecting the drug’s ability to bind to its target protein. Consequently, the NH platform can offer a more comprehensive evaluation of drug efficacy within the patient’s system, as well as valuable insights for pharmacokinetic studies. Although its nanomolar sensitivity is sufficient for measuring many biologic drugs, it remains a current limitation of the platform. Sensitivity is influenced not only by binding affinities but also by the fluorescence-based detection system. Our optimization studies have shown that higher concentrations of DNA probes significantly elevate background noise, reducing precision and compromising the signal-to-noise ratio. While achieving detection in the picomolar range remains a challenge, future optimizations will focus on refining the platform’s design and exploring innovative strategies to further enhance its sensitivity and broaden its clinical applicability. Current commercial drug monitoring systems often focus on detecting ADAs with high sensitivity but may lack clarity on the clinical relevance of the data. In contrast, the proposed approach, when further supported by clinical studies, has the potential to demonstrate the sufficiency of a single measurement for evaluating bioactive drugs, potentially surpassing existing time-consuming and labor-intensive multistep methods.

## Supporting Information Available

Supporting figures reporting data for protein–DNA conjugation, Trastuzumab dose–response assay as reference method, and detection of ADA HCA233 using the current multiepitope NH platform (PDF)

## References

[ref1] Greenberg H.; Pi-Sunyer F. X. Preventing Preventable Chronic Disease: An Essential Goal. Prog. Cardiovasc. Dis. 2019, 62, 303–305. 10.1016/j.pcad.2019.08.002. 31421079

[ref2] Tracey D.; Klareskog L.; Sasso E. H.; Salfeld J. G.; Tak P. P. Tumor Necrosis Factor Antagonist Mechanisms of Action: A Comprehensive Review. Pharmacol. Ther. 2008, 117 (2), 244–279. 10.1016/j.pharmthera.2007.10.001. 18155297

[ref3] Chimenti M. S.; Saraceno R.; Chiricozzi A.; Giunta A.; Chimenti S.; Perricone R. Profile of Certolizumab and its Potential in the Treatment of Psoriatic Arthritis. Drug Des. Devel. Ther. 2013, 7, 339–348. 10.2147/DDDT.S31658. PMC3633576 23620660

[ref4] Horiuchi T.; Mitoma H.; Harashima S.; Tsukamoto H.; Shimoda T. Transmembrane TNF-alpha: Structure, Function and Interaction with Anti-TNF Agents. Rheumatology (Oxford) 2010, 49 (7), 1215–1228. 10.1093/rheumatology/keq031. 20194223 PMC2886310

[ref5] Feldmann M.; Maini R. N. TNF Defined as a Therapeutic Target for Rheumatoid Arthritis and Other Autoimmune Diseases. Nat. Med. 2003, 9 (10), 1245–1250. 10.1038/nm939. 14520364

[ref6] Wang Y.; Liu J.; Wang Y. Role of TNF-α-induced m6A RNA Methylation in Diseases: A Comprehensive Review. Front Cell Dev. Biol. 2023, 11, 1166308 10.3389/fcell.2023.1166308. 37554306 PMC10406503

[ref7] Idriss H. T.; Naismith J. H. TNF Alpha and the TNF Receptor Superfamily: Structure-Function Relationship(s). Microsc Res. Tech 2000, 50 (3), 184–195. 10.1002/1097-0029(20000801)50:3<184::AID-JEMT2>3.0.CO;2-H. 10891884

[ref8] Taylor P. C. Pharmacology of TNF Blockade in Rheumatoid Arthritis and other Chronic Inflammatory Diseases. Curr. Opin. Pharmacol. 2010, 10 (3), 308–315. 10.1016/j.coph.2010.01.005. 20172761

[ref9] Lima M. S. R.; de Lima V. C. O.; Piuvezam G.; de Azevedo K. P. M.; Maciel B. L. L.; Morais A. H. A. Mechanisms of Action of Anti-inflammatory Proteins and Peptides with Anti-TNF-Alpha Activity and their Effects on the Intestinal Barrier: A Systematic Review. PLoS One 2022, 17 (8), e0270749 10.1371/journal.pone.0270749. 35939430 PMC9359527

[ref10] Mitoma H.; Horiuchi T.; Tsukamoto H.; Ueda N. Molecular Mechanisms of Action of Anti-TNF-α Agents - Comparison Among Therapeutic TNF-α Antagonists. Cytokine 2018, 101, 56–63. 10.1016/j.cyto.2016.08.014. 27567553

[ref11] Lim H.; Lee S. H.; Lee H. T.; Lee J. U.; Son J. Y.; Shin W.; Heo Y.-S. Structural Biology of the TNFα Antagonists Used in the Treatment of Rheumatoid Arthritis. Int. J. Mol. Sci. 2018, 19 (3), 768 10.3390/ijms19030768. 29518978 PMC5877629

[ref12] Liang S.; Dai J.; Hou S.; Su L.; Zhang D.; Guo H.; Hu S.; Wang H.; Rao Z.; Guo Y.; et al. Structural Basis for Treating Tumor Necrosis Factor α (TNFα)-associated Diseases with the Therapeutic Antibody Infliximab. J. Biol. Chem. 2013, 288 (19), 13799–13807. 10.1074/jbc.M112.433961. 23504311 PMC3650416

[ref13] Hu S.; Liang S.; Guo H.; Zhang D.; Li H.; Wang X.; Yang W.; Qian W.; Hou S.; Wang H.; et al. Comparison of the Inhibition Mechanisms of Adalimumab and Infliximab in Treating Tumor Necrosis Factor α-associated Diseases from a Molecular View. J. Biol. Chem. 2013, 288 (38), 27059–27067. 10.1074/jbc.M113.491530. 23943614 PMC3779706

[ref14] Ono M.; Horita S.; Sato Y.; Nomura Y.; Iwata S.; Nomura N. Structural Basis for Tumor Necrosis Factor Blockade with the Therapeutic Antibody Golimumab. Protein Sci. 2018, 27 (6), 1038–1046. 10.1002/pro.3407. 29575262 PMC5980524

[ref15] Lee J. U.; Shin W.; Son J. Y.; Yoo K. Y.; Heo Y. S. Molecular Basis for the Neutralization of Tumor Necrosis Factor by Certolizumab Pegol in the Treatment of Inflammatory Autoimmune Diseases. Int. J. Mol. Sci. 2017, 18 (1), 228 10.3390/ijms18010228. 28124979 PMC5297857

[ref16] Afonso J.; Lopes S.; Goncalves R.; Caldeira P.; Lago P.; Tavares de Sousa H.; Ramos J.; Goncalves A. R.; Ministro P.; Rosa I.; et al. Detection of Anti-Infliximab Antibodies is Impacted by Antibody Titer, Infliximab Level and IgG4 Antibodies: A Systematic Comparison of Three Different Assays. Therap. Adv. Gastroenterol. 2016, 9 (6), 781–794. 10.1177/1756283X16658223. PMC5076767 27803733

[ref17] Marsal J.; Barreiro-de Acosta M.; Blumenstein I.; Cappello M.; Bazin T.; Sebastian S. Management of Non-response and Loss of Response to Anti-tumor Necrosis Factor Therapy in Inflammatory Bowel Disease. Front. Med. 2022, 9, 897936 10.3389/fmed.2022.897936. PMC9241563 35783628

[ref18] Garcês S.; Demengeot J. The Immunogenicity of Biologic Therapies. Curr. Probl. Dermatol. 2018, 53, 37–48. 10.1159/000478077. 29131036

[ref19] Emi Aikawa N.; de Carvalho J. F.; Artur Almeida Silva C.; Bonfá E. Immunogenicity of Anti-TNF-alpha Agents in Autoimmune Diseases. Clin. Rev. Allergy Immunol. 2010, 38 (2–3), 82–89. 10.1007/s12016-009-8140-3. 19565360

[ref20] Vincent F. B.; Morand E. F.; Murphy K.; Mackay F.; Mariette X.; Marcelli C. Antidrug antibodies (ADAb) to Tumour Necrosis Factor (TNF)-Specific Neutralising Agents in Chronic Inflammatory Diseases: A Real Issue, a Clinical Perspective. Ann. Rheum. Dis. 2013, 72 (2), 165–178. 10.1136/annrheumdis-2012-202545. 23178294

[ref21] Pękala A.; Filip R.; Aebisher D. Anti-Drug Antibodies in Patients with Inflammatory Bowel Diseases Treated with Biosimilar Infliximab: A Prospective Cohort Study. J. Clin. Med. 2021, 10 (12), 2653 10.3390/jcm10122653. 34208676 PMC8235171

[ref22] Gaspar V. P.; Ibrahim S.; Zahedi R. P.; Borchers C. H. Utility, Promise, and Limitations of Liquid Chromatography-Mass Spectrometry-Based Therapeutic Drug Monitoring in Precision Medicine. J. Mass Spectrom. 2021, 56 (11), e4788 10.1002/jms.4788. 34738286 PMC8597589

[ref23] Rocha C.; Lago P.; Fernandes S.; Correia L.; Portela F.; Vieira A. I.; Patita M.; Arroja B.; Ministro P.; Alves C.; et al. Rapid Test Detection of Anti-Infliximab Antibodies: Performance Comparison with Three Different Immunoassays. Therap. Adv. Gastroenterol. 2020, 13, 1756284820965790 10.1177/1756284820965790. PMC7682213 33281935

[ref24] Papamichael K.; Stocco G.; Ruiz Del Agua A. Challenges in Therapeutic Drug Monitoring: Optimizing Biological Treatments in Patients with Inflammatory Bowel Disease and Other Immune-mediated Inflammatory Diseases. Ther. Drug Monit. 2023, 45 (5), 579–590. 10.1097/FTD.0000000000001095. 37012629 PMC10497208

[ref25] Porchetta A.; Ippodrino R.; Marini B.; Caruso A.; Caccuri F.; Ricci F. Programmable Nucleic Acid Nanoswitches for the Rapid, Single-Step Detection of Antibodies in Bodily Fluids. J. Am. Chem. Soc. 2018, 140 (3), 947–953. 10.1021/jacs.7b09347. 29313682

[ref26] Bertucci A.; Guo J.; Oppmann N.; Glab A.; Ricci F.; Caruso F.; Cavalieri F. Probing Transcription Factor Binding Activity and Downstream Gene Silencing in Living Cells with a DNA Nanoswitch. Nanoscale 2018, 10 (4), 2034–2044. 10.1039/C7NR07814E. 29323382

[ref27] Rossetti M.; Brannetti S.; Mocenigo M.; Marini B.; Ippodrino R.; Porchetta A. Harnessing Effective Molarity to Design an Electrochemical DNA-based Platform for Clinically Relevant Antibody Detection. Angew. Chem., Int. Ed. Engl. 2020, 59 (35), 14973–14978. 10.1002/anie.202005124. 32392398

[ref28] Fortunati S.; Pedrini F.; Del Grosso E.; Baranda Pellejero L.; Bertucci A. Design of Specific Nucleic Acid-Based Biosensors for Protein Binding Activity. Anal. Sens. 2022, 2 (6), e202200037 10.1002/anse.202200037.

[ref29] Arts R.; Ludwig S. K. J.; van Gerven B. C. B.; Estirado E. M.; Milroy L.-G.; Merkx M. Semisynthetic Bioluminescent Sensor Proteins for Direct Detection of Antibodies and Small Molecules in Solution. ACS Sens. 2017, 2 (11), 1730–1736. 10.1021/acssensors.7b00695. 29037030 PMC5706068

[ref30] Ranallo S.; Bracaglia S.; Sorrentino D.; Ricci F. Synthetic Antigen-Conjugated DNA Systems for Antibody Detection and Characterization. ACS Sens. 2023, 8 (7), 2415–2426. 10.1021/acssensors.3c00564. 37463359 PMC10391708

[ref31] Mocenigo M.; Porchetta A.; Rossetti M.; Brass E.; Tonini L.; Puzzi L.; Tagliabue E.; Triulzi T.; Marini B.; Ricci F.; et al. Rapid, Cost-Effective Peptide/Nucleic Acid-Based Platform for Therapeutic Antibody Monitoring in Clinical Samples. ACS Sens. 2020, 5 (10), 3109–3115. 10.1021/acssensors.0c01046. 32909731

[ref32] Steeland S.; Libert C.; Vandenbroucke R. E. A New Venue of TNF Targeting. Int. J. Mol. Sci. 2018, 19 (5), 1442 10.3390/ijms19051442. 29751683 PMC5983675

[ref33] Rajput A.; Ware C. F. Tumor Necrosis Factor Signaling Pathways. Encyclopedia of Cell Biology; Elsevier, 2016; pp 354–363.

[ref34] Chow S.-C. Bioavailability and Bioequivalence in Drug Development. Wiley Interdiscip. Rev. Comput. Stat. 2014, 6 (4), 304–312. 10.1002/wics.1310. 25215170 PMC4157693

[ref35] Kawano-Dourado L.; Kristianslund E. K.; Zeraatkar D.; Jani M.; Makharia G.; Hazlewood G.; Smith C.; Jess T.; Stabell C.; Schatten A.; et al. Proactive Therapeutic Drug Monitoring of Biologic Drugs in Adult Patients with Inflammatory Bowel Disease, Inflammatory Arthritis, or Psoriasis: A Clinical Practice Guideline. BMJ. 2024, 387, e079830 10.1136/bmj-2024-079830. 39467592

[ref36] Iwamoto N.; Hamada A.; Shimada T. Antibody Drug Quantitation in Coexistence with Anti-drug Antibodies on nSMOL Bioanalysis. Anal. Biochem. 2018, 540–541, 30–37. 10.1016/j.ab.2017.11.002. 29128290

