# Supplementary material for: Rapid, Single-Step Monitoring of Monoclonal Antibody Bioavailability by Using a TNF-α-Based Multiepitope DNA Nanoswitch
Source: Anal Chem. 2025 Apr 8;97(15):8195–201. doi: 10.1021/acs.analchem.5c01239 (PMC12019779; doi:10.1021/acs.analchem.5c01239)
Supplement: Supplementary file 1 — ac5c01239_si_001.pdf [file ac5c01239_si_001.pdf]

Supporting information

Rapid, Single-Step Monitoring of Monoclonal Antibody Bioavailability by Using a TNF-α-Based Multiepitope DNA Nanoswitch

Denise Di Lena[1,2,†], Edoardo Sisti[1,3,†], Erik Brass[1], Erica Belforte[4], Bruna Marini[1], Alessandro Porchetta[4], Laura Squarcia[1], Eleonora Da Pozzo[3,5], Alessandro Bertucci[2*] and Rudy Ippodrino[1*]

[1] Ulisse BioMed Labs, Area Science Park, 34149 Trieste, Italy.

[2] Department of Chemistry, Life Sciences and Environmental Sustainability, University of Parma, Parco Area delle Scienze 17/a, 43124 Parma, Italy.

[3] Department of Pharmacy, University of Pisa, via Bonanno 6, 56126 Pisa, Italy.

[4] Department of Chemistry, University of Rome, Tor Vergata, Via della Ricerca Scientifica, 00133, Rome, Italy.

[5] CISUP, Center for Instrumentation Sharing of the University of Pisa, Lungarno Pacinotti 43/44, 56126 Pisa, Italy

Supporting information

1. Supporting Figures

1. SUPPORTING FIGURES

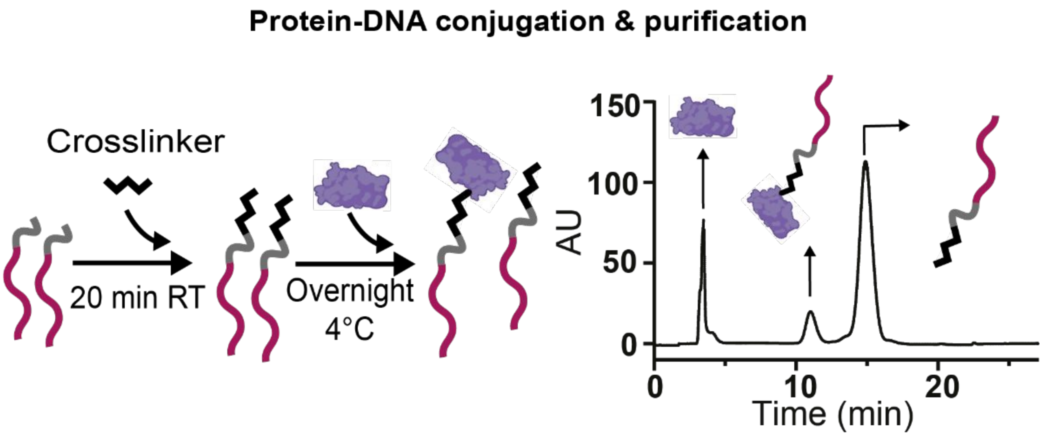

Figure S1: Protein-DNA conjugation and purification process. The conjugation reaction was performed by reacting DBCO-modified ssDNA (grey-purple strand) with the crosslinker provided in the amine-coupling kit by Dynamic Biosensors (black line), followed by overnight incubation at 4°C with TNF-α (violet protein). Purification was performed by using a proFIRE® machine (Dynamic Biosensors GmbH). The elution chromatogram report on the right obtained from the conjugation reaction mix shows three peaks: unreacted protein (left peak), conjugation product used in multiepitope nanoswitch (central peak), and excess unreacted ssDNA (right peak).

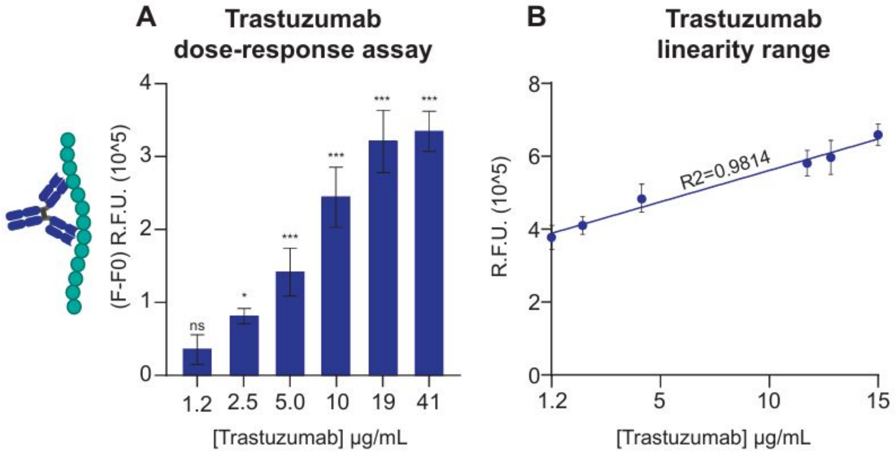

Figure S2: A) Single epitope nanoswitch dose-response assay in crude samples (undiluted blood serum) using Trastuzumab antibody as target analyte. The nanosensor demonstrated the ability to detect Trastuzumab through a peptide binding moiety at concentrations as low as 2.5 µg/mL. B) Linearity range of the Trastuzumab sensor in crude samples up to 15 µg/mL, with $R^2 = 0.9814$.

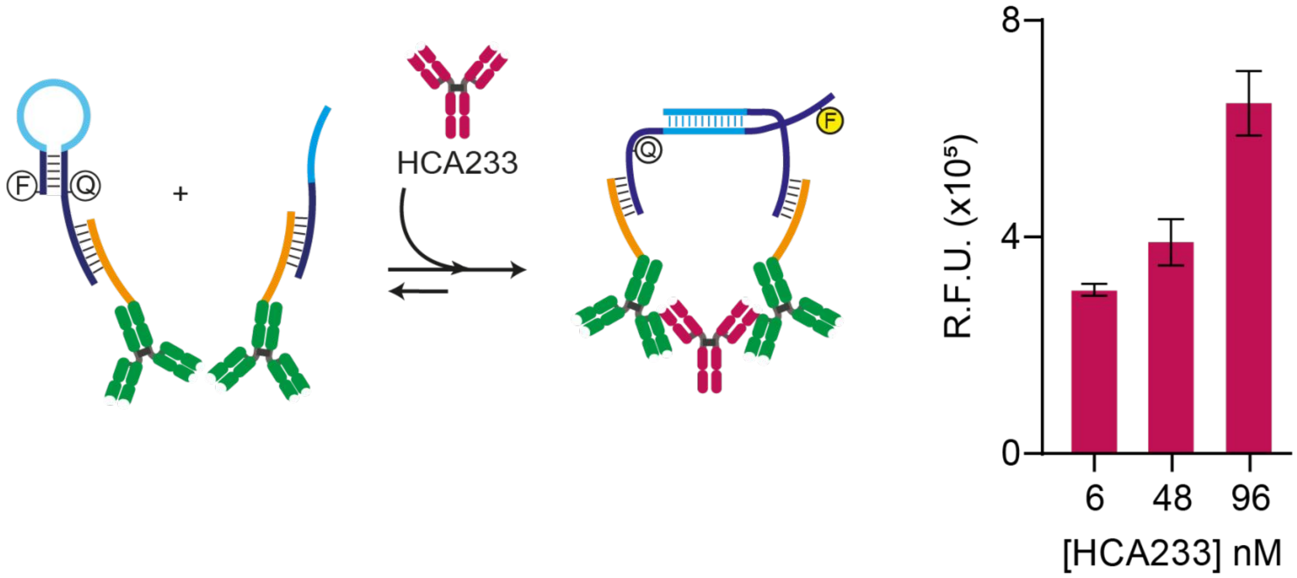

Figure S3: Detection of Anti-drug antibody (ADA) HCA233 using Infliximab as binding moiety. The system was able to detect various HCA233 concentrations up to 96 nM.
